# Association between estimated glucose disposal rate and incident cardiovascular disease in a population with Cardiovascular-Kidney-Metabolic syndrome stages 0–3: insights from CHARLS

**DOI:** 10.3389/fcvm.2025.1537774

**Published:** 2025-02-24

**Authors:** Ziyi Tan, Dayong Zhou, Yao Tang, Guijun Huo

**Affiliations:** The Affiliated Suzhou Hospital of Nanjing Medical University, Suzhou Municipal Hospital, Suzhou, Jiangsu, China

**Keywords:** Cardiovascular-Kidney-Metabolic syndrome, estimated glucose disposal rate, cardiovascular diseases, insulin resistance, CHARLS

## Abstract

**Objective:**

Estimated glucose disposal rate (eGDR) is a reliable marker of insulin resistance (IR), which has been proven to be strongly linked to cardiovascular and renal diseases. However, the link between eGDR and the occurrence of cardiovascular disease (CVD) in individuals exhibiting Cardiovascular-Kidney-Metabolic (CKM) syndrome stages 0–3 remains ambiguous.

**Methods:**

The data employed in this investigation was procured from the China Health and Retirement Longitudinal Study (CHARLS). The outcome of this study was CVD events, which include heart disease and stroke. Cox regression models and restricted cubic spline (RCS) were employed to investigate the association between eGDR and CVD risk among individuals with CKM syndrome stages 0–3.

**Results:**

This investigation encompassed 6,539 subjects, and 54.43% were female. 1,656 (26.04%) CVD events were recorded. After fully adjusting for covariates, each 1-unit increase in eGDR was linked to a 9% diminish in CVD risk (HR: 0.91, 95% CI: 0.88, 0.93). In comparison to the bottom quartile of eGDR, the adjusted HRs (95% CIs) for the second to fourth quartiles were 0.73 (95% CI: 0.64, 0.83), 0.65 (95% CI: 0.56, 0.76), and 0.56 (95% CI: 0.47, 0.66), respectively. The RCS curves revealed a substantial negative nonlinear association between eGDR and CVD events among participants with CKM syndrome stages 0–3 (*P*-value < 0.001 and *P* for nonlinear = 0.009).

**Conclusions:**

In a population with CKM stages 0–3, a significant negative nonlinear relationship was observed between eGDR and CVD risk, suggesting that eGDR might function as a useful metric for evaluating CVD risk.

## Introduction

The American Heart Association (AHA) presented the notion of Cardiovascular-Kidney-Metabolic (CKM) syndrome in 2023, and it is defined as a systemic health disorder caused by the interactions and connections between chronic kidney disease (CKD), obesity, diabetes, and cardiovascular disease (CVD) (1). Almost every organ will be affected by CKM syndrome, which will eventually lead to adverse outcomes and increased mortality. In the United States, the combined fatalities attributed to diabetes, CKD, stroke, and heart disease have exceeded 1 million in 2021, accounting for about 29 (2). In 2022, approximately 330 million residents suffer from CVD in China; the incidence and mortality rates of CVD rank first, surpassing cancer and other diseases, and CVD accounts for more than 45% of all deaths (3). In addition, the clinical impact of CKM syndrome in terms of incidence and mortality is caused by a disproportionate burden of CVD (4–7). This shows the complicated relationship between CVD, CKD, and metabolism, which should be treated as a unified system. Furthermore, the AHA considers that interventions for people with CKM stages 0–3 should focus on preventing CVD events (1). Therefore, it is urgent to develop simple and practical indicators to improve the capacity for recognizing CVD risk in people with CKM stages 0–3.

The estimated glucose disposal rate (eGDR), calculated from waist circumference, hypertension, and HbA1c, functions as a dependable indicator of insulin resistance (IR) (8, 9). Compared with hyperinsulin-glucose clamps, eGDR has higher accuracy, lower cost, and higher time efficiency, making it more suitable for clinical research (10). Recently, some clinical studies have found a connection between eGDR and CVD incidence; with the increase of baseline eGDR level, the risk of CVD is decreasing (11, 12). However, the link between eGDR and new-onset CVD in individuals with CKM syndrome stages 0–3 remains unexplored. Subjects with stages 0–3 of CKM syndrome were evaluated for the link between eGDR and CVD risk in this investigation.

To address this knowledge gap, subjects were enlisted from the China Health and Retirement Longitudinal Study (CHARLS). This investigation sought to explore the link between the baseline eGDR level and CVD risk in people with CKM syndrome stages 0–3 and provide more evidence on the practical application of the eGDR in the real world.

## Methods

### Study population

The information for this cohort investigation comes from CHARLS. CHARLS is a national longitudinal survey aimed at middle-aged and elderly families and individual residents aged 45 and older in China (13). The baseline national wave of CHARLS was executed in 2011, encompassing approximately 10,000 households and 17,708 participants across 150 counties/districts and 450 villages/resident committees. Follow-up assessments of the participants occur every two to three years. Five waves of survey data (2011–2020) have been published. The Institutional Review Board at Peking University authorized this investigation, and all subjects submitted signed consent forms prior to participation. The research conformed to the Strengthening the Reporting of Observational Studies in Epidemiology (STROBE) recommendations (14).

**Figure 1:**
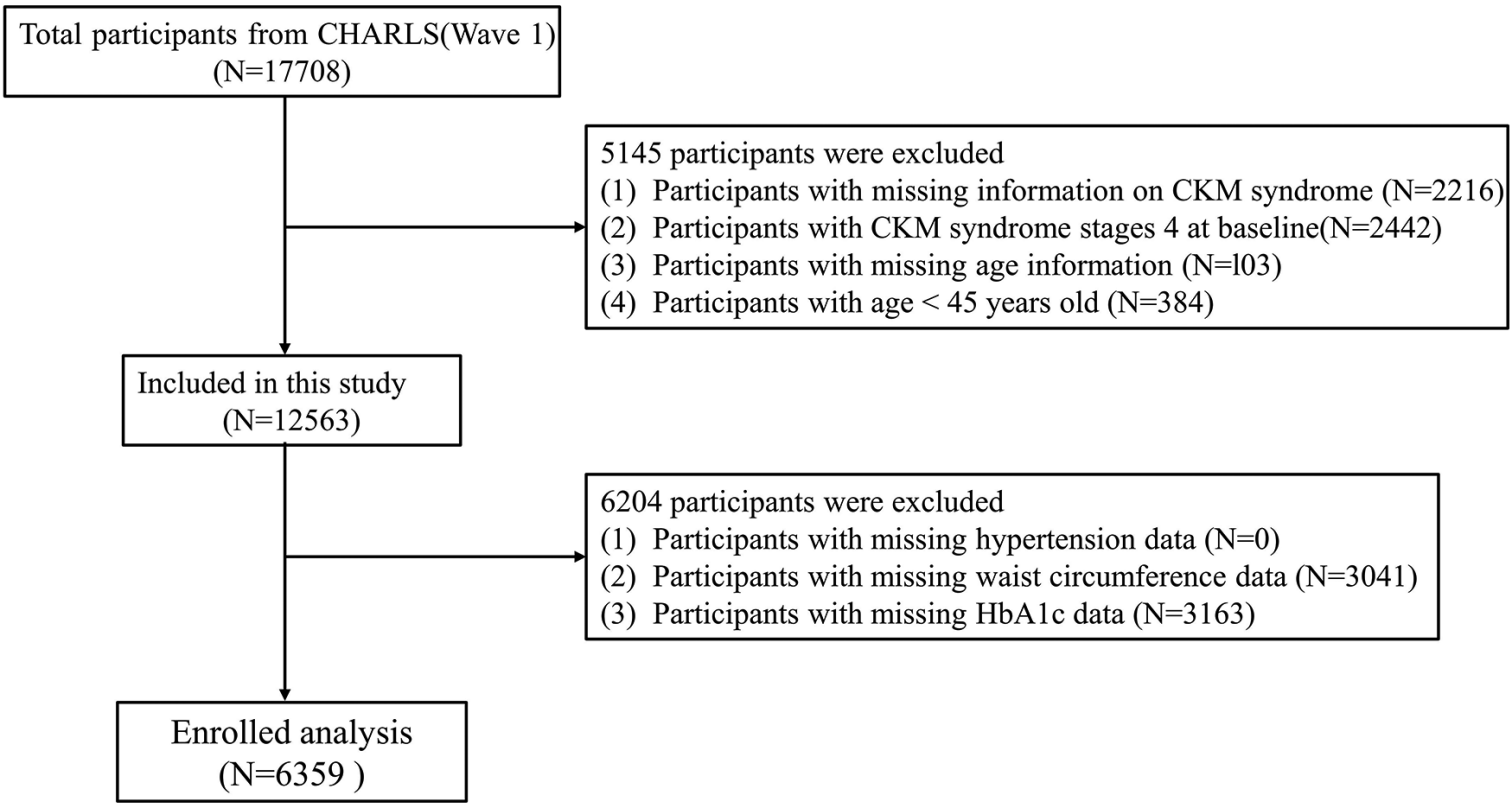
The flowchart of study participants.

The screening process of subjects is depicted in Figure 1. In this investigation, 17,708 participants in 2011 were the baseline data, with subsequent follow-ups in 2013, 2015, 2018, and 2020. Initially, 2,216 participants were missing information on CKM syndrome at baseline, and 2,442 participants with CKM syndrome stage 4 were excluded. We also excluded 103 participants lacking age information and 384 participants aged <45 years old. Furthermore, we removed 0 participants who were missing hypertension data, 3,041 participants who were missing waist circumference data, and 3,163 participants who lacked HbA1c data. Consequently, 11,349 individuals were omitted from the study, while 6,539 subjects were incorporated into the final analysis.

### Estimation of eGDR

The eGDR was computed utilizing the formula: eGDR = 21.158 − (0.09 × WC) − (3.407 × HT) − (0.551 × HbA1c), where WC is waist circumference (cm), HT is hypertension status (yes = 1, no = 0) (11).

### Assessment of incident CVD

CVD encompassed both heart disease and stroke, which were evaluated through two key questions: (1) “Have you ever been diagnosed by a physician with heart attack, coronary artery disease, angina, heart failure, or any other cardiac conditions?” (2) “Have you ever been diagnosed with stroke by a physician?”. The CVD onset period was defined as the interval between the last interview and the one in which the CVD was first recorded (15, 16).

### Definition of CKM syndrome

The phases of CKM syndrome are categorized according to the AHA Presidential Advisory Statement on CKM syndrome (1). The progression of CKM syndrome is as follows: Stage 0, no CKM risk factors; Stage 1, surplus or impaired adiposity; Stage 2, metabolic risk factors and CKD; Stage 3, subclinical CVD in CKM; Stage 4: Clinical CVD in CKM. Subclinical CVD was defined as having ≥20% of 10-year CVD risk or high-risk CKD by the AHA Predicting Risk of CVD Events (PREVENT) equations (17).

### Assessments of covariates

The data of participants at baseline was collected by trained interviewers using structured questionnaires. (1) Demographic and lifestyle data: gender, age, residence (classified as rural and urban), education level, marital status (married or other), smoking and drinking status (classified as never, former, and current). (2) Body measurements: weight, WC, systolic blood pressure (SBP), and diastolic blood pressure (DBP). (3) Information on Disease and medication history: lung diseases, liver diseases, cancer, hypertension, diabetes, dyslipidemia, hypertension medication, dyslipidemia medication, and diabetes medication. (4) Laboratory test data: platelet, c-reactive protein (CRP), blood urea nitrogen (BUN), fasting blood glucose (FBG), serum creatinine (Scr), total cholesterol (TC), triglycerides (TG), high-density lipoprotein cholesterol (HDL-C), low-density lipoprotein cholesterol (LDL-C), uric acid (UA), and HbA1c.

Hypertension was marked by fulfilling at least one of the subsequent conditions: (1) SBP ≥ 140 mmHg, (2) DBP ≥ 90 mmHg, (3) current usage of antihypertensive medications, or (4) self-reported hypertension diagnosed by a physician. Diabetes was identified by an FBG ≥ 126 mg/dl, utilization of antidiabetic medications, or self-reported diabetes diagnosed by a physician. Any of the following marked Dyslipidemia: (1) TG ≥ 150 mg/dl, (2) TC ≥ 240 mg/dl, (3) HDL-C < 40 mg/dl, (4) LDL-C ≥ 160 mg/dl, (5) current consumption of lipid-lowering drugs, or (6) self-declared dyslipidemia diagnosed by a physician. Metabolic syndrome (MetS) was marked by fulfilling three or more of the following criteria: (1) SBP ≥ 130 mmHg or DBP ≥ 80 mmHg and/or utilization of antihypertensive medications; (2) FBG ≥ 100 mg/dl; (3) TG ≥ 150 mg/dl; (4) HDL-C < 40 mg/dl for male and <50 mg/dl for female; and (5) WC ≥ 80 cm for female and ≥90 cm for male (1). Considering that this study was conducted on a Chinese population, the Chinese Modification of Diet in Renal Disease (C-MDRD) equation was employed to ascertain the estimated glomerular filtration rate (eGFR) (18). It was categorized into CKD stages per the Kidney Disease Improving Global Outcomes (KDIGO) (1).

### Statistical analysis

The extent of data missing in this study is shown in Supplementary Table S1. Although only a small amount of data was missing, we still used multiple imputations to impute the missing values to mitigate potential bias (19). Normally distributed quantitative variables were denoted as mean ± standard error, and group differences were inferred utilizing analysis of variance (ANOVA). Non-normally distributed quantitative variables were denoted as medians and interquartile ranges, and the Kruskal–Wallis test was applied to infer disparities among cohorts. Categorical variables were reported as counts and proportions and assessed utilizing the Chi-squared test.

Subjects were categorized into four cohorts grounded in baseline eGDR values. Quartile 1 (Q1), eGDR ≤ 6.966; Quartile 2 (Q2), 6.966 < eGDR ≤ 8.695; Quartile 3 (Q3), 8.695 < eGDR ≤ 10.753; Quartile 4 (Q4), eGDR > 10.753. In addition, to improve result reliability, eGDR was also analyzed as a continuous variable. Kaplan–Meier curves, accompanied by the log-rank test, were utilized to estimate the cumulative CVD incidence. Collinearity between eGDR and other covariates was assessed through tolerance values and variance inflation factors (VIFs) (20). The outcomes indicated that the VIF for TC, TG, and LDL-C was greater than 5, leading to their exclusion from the multivariate model (Supplementary Table S2). Cox proportional hazard regression models were utilized to investigate the link between eGDR and CVD risk, and hazard ratios (HRs) with 95% confidence intervals (95%CIs) were calculated. Three models were developed with varying levels of covariate adjustment. Model 1 remained unmodified, Model 2 incorporated modifications for gender, age, residence, marital status, education level, smoking status, and drinking status, and Model 3 additionally refined for diabetes, dyslipidemia, diabetes medications, dyslipidemia medications, platelets, CRP, BUN, FBG, Scr, HDL-C, UA, BMI, SBP, and DBP. Moreover, the fully adjusted restricted cubic splines (RCS) analysis was carried out to analyze the dose-response link between eGDR and CVD risk in individuals with CKM syndrome stages 0–3. To examine the link between eGDR and CVD occurrence in people at different CKM syndrome stages, CKM stage 2 and CKM stage 3 cohorts were also analyzed using the RCS curve.

Furthermore, subgroup analyses were carried out to explore whether the relationship between eGDR and CVD risk was consistent across various demographics, encompassing gender, age, residence, hypertension, diabetes, dyslipidemia, smoking status, drinking status, BMI, and CKM syndrome stages. Interaction analyses were also conducted to examine potential modifications of CVD risk across these subgroups. To ensure the stability of the study findings, we implemented three sensitivity analyses. Firstly, eGDR was recalculated to align with the redefined hypertension threshold (130/80 mmHg). Secondly, the data were reanalyzed after excluding all missing values. Thirdly, E-values were calculated based on Model 3 to assess the minimum strength of the link between unmeasured confounders and eGDR, which could explain the observed relationship with CVD risk (21). All statistical analyses were executed employing Stata 17.0 and R version 4.2.2, with *P*-values < 0.05 deemed statistically significant.

## Results

### Baseline characteristics of participants

After exclusion, our final analysis incorporated 6,359 participants from the CHARLS, with a mean age of 59.61 ± 9.49 years. The participants of our investigation included 2,898 (45.57%) males and 3,461 (54.43%) females. Comparing individuals in the highest and lowest eGDR quartiles suggested that those with elevated eGDR levels were more prone to be former drinkers and non-hypertensive. The differences were also observed in laboratory tests; higher eGDR quartiles were linked to decreased Scr, TC, and UA. The baseline information of all participants was described across eGDR quartiles in Table 1.

**Table 1:** Baseline characteristics stratified by eGDR quartiles.

Characteristic	eGDR quartiles					*p*-value
	Overall	Q1	Q2	Q3	Q4	
No. of subjects	6,359	1,586	1,593	1,590	1,590	
Gender						0.002
Female	3,461 (54.43%)	912 (57.50%)	818 (51.35%)	890 (55.97%)	841 (52.89%)	
Male	2,898 (45.57%)	674 (42.50%)	775 (48.65%)	700 (44.03%)	749 (47.11%)	
Age, year	59.61 ± 9.49	60.94 ± 9.30	61.93 ± 9.86	57.70 ± 8.91	57.86 ± 9.13	<0.001
Residence						<0.001
Rural	4,140 (65.10%)	935 (58.95%)	1,082 (67.92%)	991 (62.33%)	1,132 (71.19%)	
Urban	2,219 (34.90%)	651 (41.05%)	511 (32.08%)	599 (37.67%)	458 (28.81%)	
Marital status						<0.001
Marred	5,528 (86.93%)	1,375 (86.70%)	1,305 (81.92%)	1,433 (90.13%)	1,415 (88.99%)	
Other	831 (13.07%)	211 (13.30%)	288 (18.08%)	157 (9.87%)	175 (11.01%)	
Education level						<0.001
No formal education	3,164 (49.76%)	784 (49.43%)	862 (54.11%)	728 (45.79%)	790 (49.69%)	
Primary school	1,378 (21.67%)	343 (21.63%)	356 (22.35%)	338 (21.26%)	341 (21.45%)	
Middle school	1,224 (19.25%)	302 (19.04%)	270 (16.95%)	347 (21.82%)	305 (19.18%)	
High school or above	593 (9.33%)	157 (9.90%)	105 (6.59%)	177 (11.13%)	154 (9.69%)	
Smoking status						<0.001
Never	3,907 (61.44%)	1,042 (65.70%)	923 (57.94%)	1,011 (63.58%)	931 (58.55%)	
Former	529 (8.32%)	164 (10.34%)	129 (8.10%)	147 (9.25%)	89 (5.60%)	
Current	1,923 (30.24%)	380 (23.96%)	541 (33.96%)	432 (27.17%)	570 (35.85%)	
Drinking status						<0.001
Never	3,894 (61.24%)	964 (60.78%)	929 (58.32%)	1,013 (63.71%)	988 (62.14%)	
Former	509 (8.00%)	176 (11.10%)	147 (9.23%)	102 (6.42%)	84 (5.28%)	
Current	1,956 (30.76%)	446 (28.12%)	517 (32.45%)	475 (29.87%)	518 (32.58%)	
Hypertension	3,224 (50.70%)	1,579 (99.56%)	1,473 (92.47%)	125 (7.86%)	47 (2.96%)	<0.001
Diabetes	1,393 (21.91%)	473 (29.82%)	305 (19.15%)	335 (21.07%)	280 (17.61%)	<0.001
Dyslipidemia	3,653 (57.45%)	1,058 (66.71%)	752 (47.21%)	989 (62.20%)	854 (53.71%)	<0.001
Lung disease	559 (8.79%)	131 (8.26%)	162 (10.17%)	117 (7.36%)	149 (9.37%)	0.028
Liver disease	174 (2.74%)	40 (2.52%)	40 (2.51%)	54 (3.40%)	40 (2.52%)	0.325
Cancer	52 (0.82%)	17 (1.07%)	9 (0.56%)	13 (0.82%)	13 (0.82%)	0.472
Hypertension medications	1,351 (21.25%)	802 (50.57%)	499 (31.32%)	33 (2.08%)	17 (1.07%)	<0.001
Diabetes medications	262 (4.12%)	136 (8.58%)	58 (3.64%)	53 (3.33%)	15 (0.94%)	<0.001
Dyslipidemia medications	283 (4.45%)	151 (9.52%)	59 (3.70%)	44 (2.77%)	29 (1.82%)	<0.001
Platelets, (10^9^/L)	213.78 ± 72.61	217.23 ± 72.61	211.50 ± 75.42	212.28 ± 70.39	214.11 ± 71.83	0.119
CRP, mg/dl	1.13 (0.59, 2.39)	1.58 (0.82, 3.01)	1.05 (0.55, 2.34)	1.11 (0.61, 2.20)	0.85 (0.48, 1.95)	<0.001
BUN, mg/dl	15.64 ± 4.46	15.67 ± 4.43	15.94 ± 4.73	15.48 ± 4.42	15.49 ± 4.23	0.013
Scr, mg/dl	0.78 ± 0.24	0.80 ± 0.21	0.80 ± 0.34	0.77 ± 0.19	0.76 ± 0.18	<0.001
FBG, mg/dl	104.22 (95.58, 116.73)	107.95 (98.82, 123.93)	102.78 (94.86, 114.66)	104.40 (95.76, 117.00)	102.06 (94.14, 112.14)	<0.001
TC, mg/dl	195.03 ± 39.63	202.04 ± 40.39	195.01 ± 36.84	194.43 ± 39.90	188.64 ± 40.17	<0.001
TG, mg/dl	122.13 (83.19, 175.23)	138.06 (96.46, 198.02)	105.32 (74.34, 153.99)	129.21 (89.39, 184.43)	115.49 (77.88, 162.84)	<0.001
HDL-C, mg/dl	48.55 ± 14.72	45.80 ± 13.36	52.99 ± 15.76	45.86 ± 13.53	49.55 ± 14.89	<0.001
LDL-C, mg/dl	116.56 ± 36.54	120.89 ± 38.56	115.93 ± 35.64	116.79 ± 35.98	112.63 ± 35.46	<0.001
HbA1c, %	5.10 (4.90, 5.50)	5.30 (5.00, 5.70)	5.10 (4.80, 5.40)	5.20 (4.90, 5.50)	5.00 (4.80, 5.30)	<0.001
UA, mg/dl	4.51 ± 1.27	4.75 ± 1.33	4.50 ± 1.26	4.49 ± 1.24	4.29 ± 1.21	<0.001
eGFR ml/min/1.73 m^2^	120.91 ± 31.33	116.86 ± 33.34	119.35 ± 31.21	122.55 ± 30.69	124.88 ± 29.40	<0.001
Height, m	1.58 ± 0.09	1.58 ± 0.09	1.57 ± 0.10	1.58 ± 0.10	1.57 ± 0.09	<0.001
Weight, kg	59.54 ± 11.74	67.05 ± 11.41	55.58 ± 9.95	62.32 ± 10.57	53.22 ± 9.53	<0.001
Waist, cm	85.32 ± 12.43	95.82 ± 6.88	82.22 ± 7.28	89.17 ± 8.43	74.09 ± 13.66	<0.001
BMI, kg/m^2^	23.53 (21.23, 26.16)	26.57 (24.53, 28.73)	22.17 (20.45, 24.01)	24.66 (22.81, 26.73)	21.32 (19.72, 23.03)	<0.001
MetS	2,257 (35.49%)	851 (53.66%)	351 (22.03%)	739 (46.48%)	316 (19.87%)	<0.001
SBP	135.43 ± 21.08	148.61 ± 19.35	146.93 ± 19.89	124.42 ± 14.39	121.77 ± 13.65	<0.001
DBP	78.33 ± 11.84	84.26 ± 11.61	82.89 ± 11.94	73.91 ± 9.06	72.26 ± 9.44	<0.001
CKM stage						<0.001
Stage 0	107 (1.68%)	0 (0.00%)	0 (0.00%)	5 (0.31%)	102 (6.42%)	
Stage 1	280 (4.40%)	0 (0.00%)	8 (0.50%)	131 (8.24%)	141 (8.87%)	
Stage 2	5,205 (81.85%)	1,309 (82.53%)	1,429 (89.70%)	1,263 (79.43%)	1,204 (75.72%)	
Stage 3	767 (12.06%)	277 (17.47%)	156 (9.79%)	191 (12.01%)	143 (8.99%)	

### Associations of eGDR with incident CVD in individuals with CKM syndrome stages 0–3

**Figure 2:**
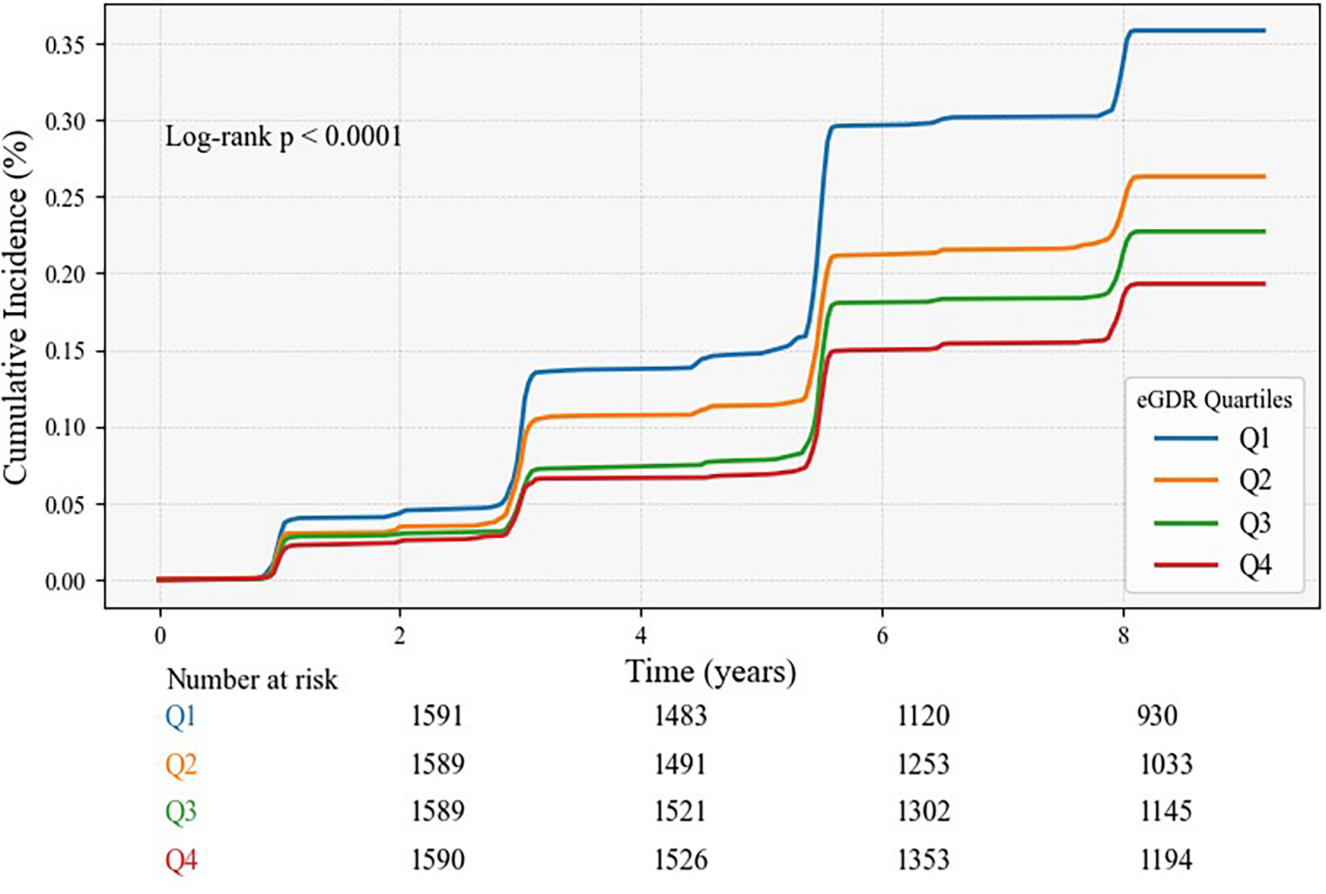
The Kaplan–Meier analysis for the cumulative incidence of CVD was based on eGDR quartiles for total participants.

A sum of 1,656 (26.04%) participants experienced a CVD. CVD prevalence decreased progressively from Q1 to Q4, with 567 (8.92%) in Q1, 421 (6.62%) in Q2, 361(5.68%) in Q3, and 307 (4.83%) in Q4. Kaplan–Meier curves demonstrated a gradual reduction in CVD incidence from Q1 to Q4, with statistical significance (Log-rank test *P* < 0.001) (Figure 2). The Cox proportional hazard models confirmed a notable link between eGDR and CVD risk. In Model 1, a Per 1-unit increase in eGDR was linked to an 11% diminish in CVD risk (HR: 0.89, 95% CI: 0.87, 0.91). Model 2 suggested that each 1-unit increment in eGDR corresponded to an 11% reduction in CVD risk (HR: 0.89, 95% CI: 0.87, 0.91). Model 3 demonstrated a 9% decline in risk for every 1-unit elevation in eGDR (HR: 0.91, 95% CI: 0.88, 0.93).

Furthermore, eGDR was categorized into quartiles to examine its association with CVD occurrence. After full covariate adjustment (Model 3), the adjusted HRs (95% CIs) for Q2, Q3, and Q4, in contrast to Q1, were 0.73 (95% CI: 0.64, 0.83), 0.65 (95% CI: 0.56, 0.76), and 0.56 (95% CI: 0.47, 0.66), respectively. These findings indicated that subjects in Q2, Q3, and Q4 exhibited a 27%, 35%, and 44% reduced CVD risk relative to those in Q1 (Table 2).

**Table 2:** Association between the eGDR and CVD incidence in a population with CKM syndrome stages 0–3.

Characteristic	Event, *n*	Model 1		Model 2		Model 3	
		HR (95% CI)	*p*-value	HR (95% CI)	*p*-value	HR (95% CI)	*p*-value
eGDR (per 1 unit)	1,656	0.89 (0.87, 0.91)	<0.001	0.89 (0.87, 0.91)	<0.001	0.91 (0.88, 0.93)	<0.001
eGDR quartile							
Q1	567	Ref		Ref		Ref	
Q2	421	0.70 (0.61, 0.79)	<0.001	0.70 (0.62, 0.80)	<0.001	0.73 (0.64, 0.83)	<0.001
Q3	361	0.58 (0.51, 0.66)	<0.001	0.60 (0.53, 0.69)	<0.001	0.65 (0.56, 0.76)	<0.001
Q4	307	0.48 (0.42, 0.56)	<0.001	0.51 (0.44, 0.59)	<0.001	0.56 (0.47, 0.66)	<0.001
*P* for trend			<0.001		<0.001		<0.001

HR, hazard ratio; CI, confidence interval.

Model 1: unadjusted for any covariates.

Model 2: adjusted for gender, age, residence, marital status, education level, smoking status, and drinking status.

Model 3: adjusted for gender, age, residence, marital status, education level, smoking status, drinking status, diabetes, dyslipidemia, diabetes medications, dyslipidemia medications, platelets, CRP, BUN, FBG, Scr, HDL-C, UA, BMI, SBP, and DBP.

**Figure 3:**
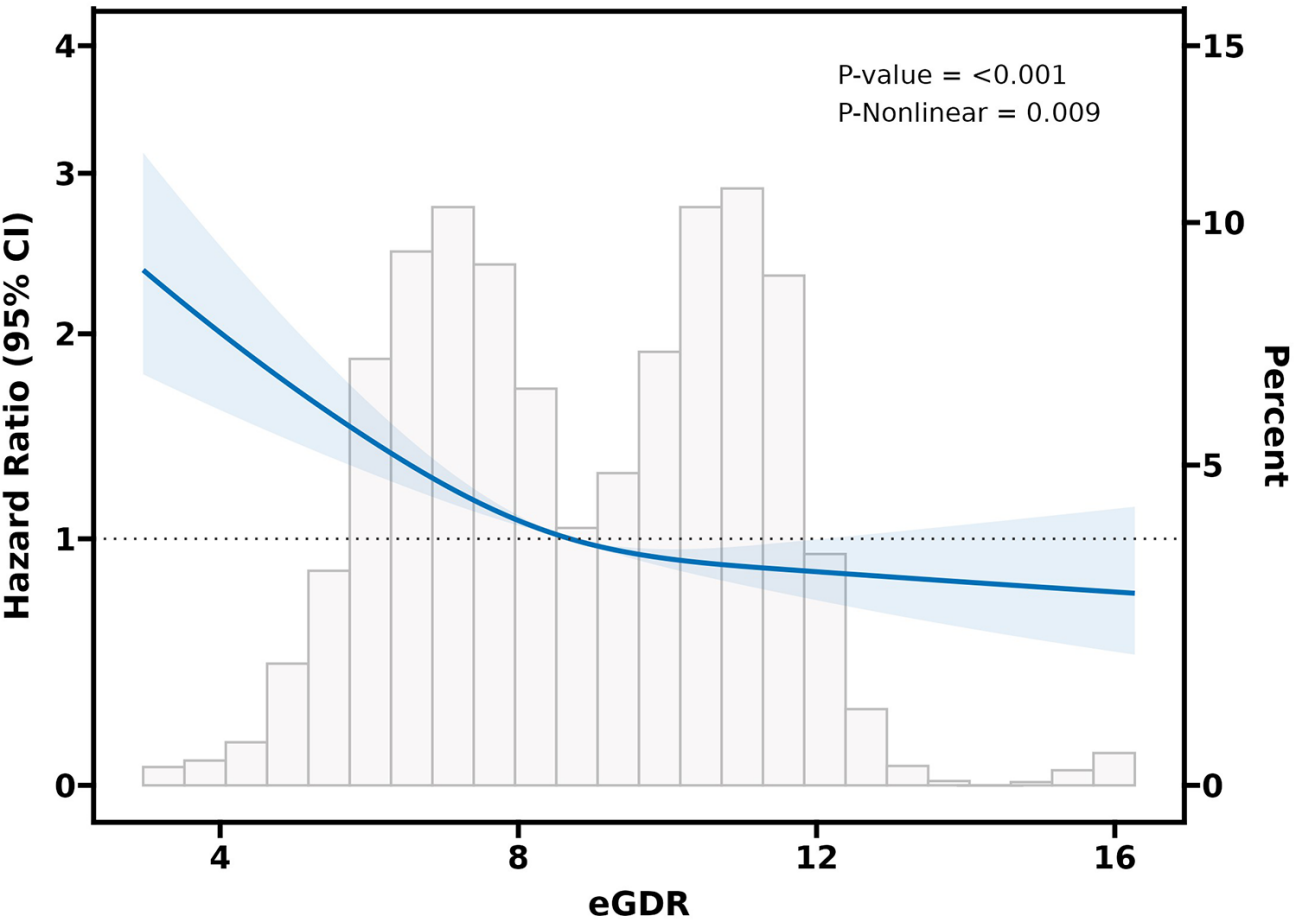
Association of eGDR and the risk of CVD in a population with CKM syndrome stages 0-3 using a multivariable-adjusted RCS model. The model was adjusted for gender, age, residence, marital status, education level, smoking status, drinking status, diabetes, dyslipidemia, diabetes medications, dyslipidemia medications, platelets, CRP, BUN, FBG, Scr, HDL-C, UA, BMI, SBP, and DBP.

**Figure 4:**
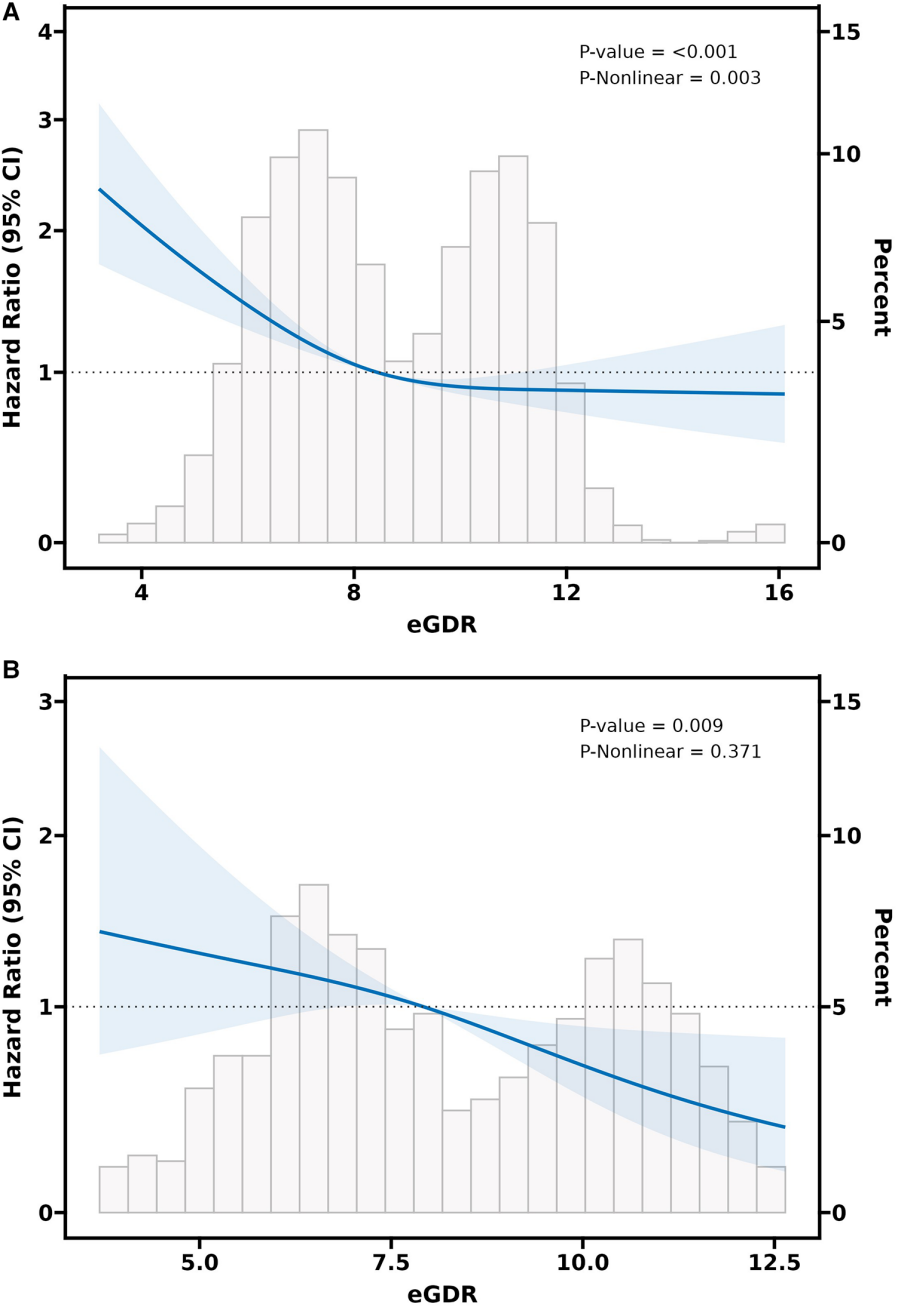
The RCS analysis between the eGDR and CVD incidence in a population with CKM syndrome stage 2 **(A)** or stage 3 **(B)**. The model was adjusted for gender, age, residence, marital status, education level, smoking status, drinking status, diabetes, dyslipidemia, diabetes medications, dyslipidemia medications, platelets, CRP, BUN, FBG, Scr, HDL-C, UA, BMI, SBP, and DBP.

Adjusted RCS curves (Model 3) revealed a significant negative nonlinear relationship between eGDR and CVD events among participants with CKM syndrome stage 0–3 (*P*-value < 0.001 and *P* for nonlinear = 0.009) (Figure 3). Likewise, in subjects with CKM syndrome stage 2, a significant negative nonlinear connection was also evident between the eGDR and CVD risk (*P*-value < 0.001 and *P* for nonlinear = 0.003) (Figure 4A). Nonetheless, in individuals with CKM syndrome stage 3, this examination indicated a marked inverse linear dose-response link between eGDR and CVD risk (*P*-value = 0.009 and *P* for nonlinear = 0. 371) (Figure 4B). Further research shows that there is a significant negative linear relationship between cumulative eGDR and CVD events among participants with CKM syndrome grade 0–3 (Supplementary Figures S1 and S2; Supplementary Table S3).

### Subgroup analyses

**Figure 5:**
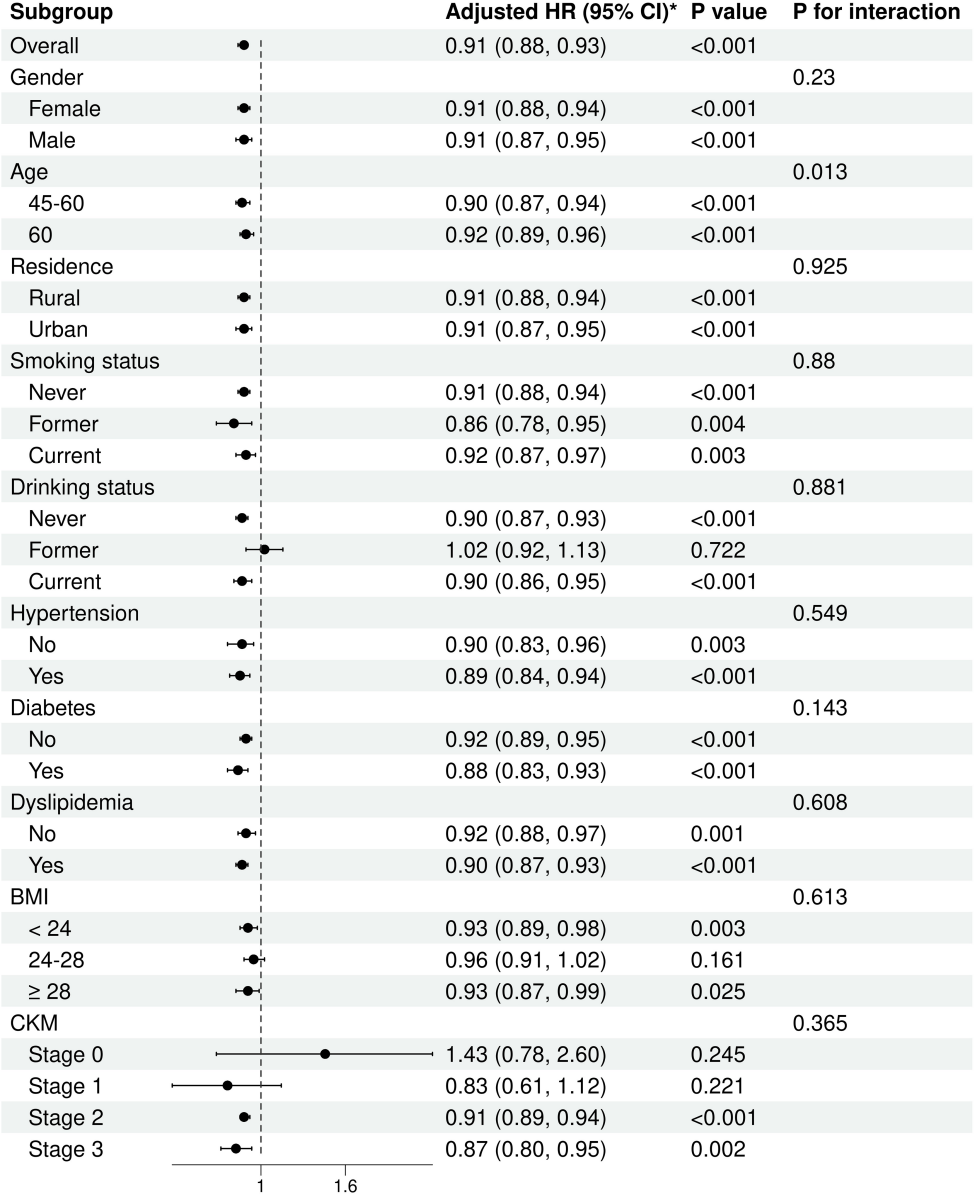
Subgroup and interaction analyses of the association between the eGDR and CVD incidence in a population with CKM syndrome stages 0-3. The model was adjusted for gender, age, residence, marital status, education level, smoking status, drinking status, diabetes, dyslipidemia, diabetes medications, dyslipidemia medications, platelets, CRP, BUN, FBG, Scr, HDL-C, UA, BMI, SBP, and DBP.

To further explore the link between baseline eGDR and CVD occurrence, subgroup analyses were performed across diverse demographic characteristics, including gender, age, residence, smoking status, drinking status, hypertension, diabetes, dyslipidemia, BMI, and CKM syndrome stage. The findings suggested that higher eGDR values were associated with lower incidence of CVD, which was consistent across different subgroups, encompassing gender, age, residence, smoking status, never drinkers, current drinkers, hypertension, diabetes, dyslipidemia, BMI < 24, BMI ≥ 28, and CKM syndrome stage 2–3. However, this association was not observed in the former drinkers, 24 ≤ BMI <28, and CKM syndrome stage 0–1. Interaction analyses were also executed to explore possible interplay between these subgroups and eGDR. The results indicated that significant interactions were noted between eGDR and age (*P* for interaction = 0.013). Nevertheless, no notable interplay was observed between eGDR and other subgroups (Figure 5).

### Sensitivity analysis

To assess the stability of the outcomes, we conducted three sensitivity analyses. The findings remained largely unaltered when hypertension was redefined (130/80 mmHg) (Supplementary Tables S4 and S5). Additionally, after removing all missing data and reanalyzing, there is no noticeable change in the final research results (Supplementary Table S6 and S7). Furthermore, the E-value for eGDR was calculated based on Model 3, revealing a value of 1.43, indicating that only a relatively large unmeasured confounding factor could explain the observed association.

## Discussion

This research represents the initial large-scale investigation to establish a marked association between eGDR and CVD risk in individuals with CKM syndrome stages 0–3. The eGDR levels were strongly correlated with reduced CVD risk, a connection that persisted markedly even after fully adjusting for covariates. Additionally, the RCS regression model demonstrated a substantial inverse nonlinear relationship between eGDR and CVD risk. They proved the consistency of the link between eGDR and CVD in different populations. Furthermore, subgroup analyses and interaction analysis proved the consistency of the link between eGDR and CVD risk across various demographics. Subsequently, the robustness of the findings was confirmed through three sensitivity analyses, including redefining hypertension, removing missing data, and calculating the E-value, all of which supported the strength and reliability of the observed associations.

CVD, CKD and metabolic diseases were widespread in the population. According to a research report of 11,607 adults, 26.3% suffered from at least one ailment, 8.0% suffered from at least two, and 1.5% grappled with all three conditions simultaneously (22). CVD, CKD and metabolic diseases affect people's health together and cause a heavy burden to the public health system (23). To reduce the incidence of CVD and the burden on the medical and health system, it is necessary to carry out comprehensive prevention and systematic management of patients (24, 25). Therefore, the AHA has recently consistently defined CKM syndrome as a systemic disease (1). IR is characterized by reduced sensitivity to insulin's physiological effects, which is a crucial risk element for atherosclerosis (26–28). IR is broadly acknowledged as a major factor in CVD and mortality (29). The eGDR is a reliable indicator for evaluating IR, and its predictive role in CVD has been demonstrated (30). Patients with CKM syndrome need effective biomarkers for early detection and treatment. Therefore, this investigation examines the complex link between eGDR and CVD risk in individuals with CKM syndrome.

This study primarily focused on the general population aged 45 and older in China, enrolling 6,359 participants from a nationwide prospective cohort. The findings indicated a notable correlation between baseline eGDR levels and CVD events. With each single-unit rise in eGDR, the likelihood of CVD occurrence diminished by 9%. In contrast to the lowest eGDR quartile, individuals in the highest eGDR quartile of CKM syndrome exhibited a 44% reduced risk of CVD. More importantly, our study revealed a significant negative nonlinear relationship between eGDR and CVD events among subjects with CKM syndrome stages 0–3. These results show the predictive value of eGDR in the CKM syndrome population, and clinicians should dynamically monitor the level of eGDR to identify high-risk individuals who may develop CVD more accurately.

The findings from the subgroup analyses suggested no statistically meaningful correlation between eGDR and CVD occurrence in subjects with CKM syndrome stages 0 and 1. According to the definition of CKM syndrome stages given by AHA, no CKM Risk factors are defined as CKM syndrome stage 0, and excess or impaired adiposity is defined as CKM syndrome stage 1 (1). Therefore, these participants have no or only a few cardiovascular risk factors. Furthermore, the study sample was drawn from the CHARLS database and thus consisted solely of middle-aged and elderly individuals, potentially introducing some bias. Therefore, it may be these two reasons that no significant correlation between eGDR and CVD was observed in the participants of CKM syndrome stages 0 and 1. On the contrary, there is substantial statistical significance between eGDR and CVD incidence in subjects with high metabolic risk factors in CKM syndrome stages 2 and 3. On the contrary, among participants with high cardiovascular risk factors at CKM syndrome stages 2 and 3, there is significant statistical significance between eGDR and CVD incidence. Furthermore, the link between eGDR and CVD risk was magnified in individuals with CKM syndrome stage 3, and the results showed a 13% risk reduction for each unit increase in eGDR. Moreover, in participants aged 45–60 years, every unit rise in eGDR corresponded to a 10% decrease in CVD risk. Participants aged older than 60 years showed an 8% decrease per unit eGDR increase, and significant interactions were noted between eGDR and age. This suggests that controlling eGDR levels in people aged 45–60 years could markedly reduce CVD incidence relative to people over 60 years of age. This requires clinicians to pay more attention to the population aged 45–60 years with CKM syndrome stages 0–3. There is no significant difference in the link between eGDR and CVD incidence in another variable, which indicates that our research results are universal to a wide range of people.

While the exact pathway connecting eGDR to CVD in individuals with CKM syndrome is still unclear, several potential explanations exist. Initially, eGDR is a reliable marker for evaluating IR, which may disrupt glucose metabolism equilibrium. Leading to decreased insulin sensitivity, which is a known risk factor for various cardiovascular diseases (12, 31). This disturbance subsequently initiates inflammatory processes and oxidative stress and eventually leads to the formation of atherosclerosis, chronic inflammation and atherosclerosis are important factors in the development of CVD (32–35). Additionally, in IR, insulin's action is shifted towards vasoconstriction, hypertrophy of smooth muscle cells and accelerated atherosclerosis via activation of the MAPK pathway. Thereby compromising vascular endothelial function and leading to vascular damage, impairs the utilization of nitric oxide, which will markedly increase the incidence of CVD (36–38). Moreover, IR can lead to an increase in visfatin levels, and visfatin favors proinflammatory cytokine production and inhibits insulin signaling via the signal transducer and activator of transcription 3(STAT3) and NF-kB pathways, thus rendering it a valuable predictor of metabolic disturbances and CVD events (39, 40). Furthermore, IR is related to a series of metabolic abnormalities, encompassing hypertension, glucose intolerance, hypertension, dyslipidemia, and so on, which are collectively called insulin resistance syndrome (IRS) (41–43). Every factor of IRS is an RF for cardiovascular events, which promotes the growth and proliferation of vascular smooth muscle, inflammation, and atherosclerosis, eventually leading to CVD events (44–46). Therefore, patients with IR may have underlying vascular and organ damage, increasing the risk of CVD. In clinical practice, recognizing the link between IR, represented by eGDR, and CVD risk can enhance overall risk assessment, enabling clinicians to make more informed decisions and develop personalized treatment and management strategies.

This investigation presents several notable merits. Firstly, this prospective, extensive cohort analysis represents the initial exploration of eGDR's connection to CVD among individuals exhibiting CKM syndrome stages 0–3. Secondly, we employed the most recent PREVENT equation to characterize subclinical CVD. Thirdly, this study not only analyzed eGDR as a continuous variable but also analyzed it as a categorical variable to evaluate its relationship with CVD at different levels. Fourthly, the RCS curve was used to identify a significant negative nonlinear relationship between eGDR and CVD risk; it provides a visual representation so clinicians can better understand the link between eGDR and CVD risk. Fifthly, subgroup analyses and interaction analysis were performed in this study, providing valuable insights for clinicians. Finally, we also performed three sensitivity analyses to demonstrate the reliability of these observations.

However, several limitations must be acknowledged. Firstly, the study relied on baseline eGDR data; incorporating dynamic eGDR data would enhance CVD risk stratification. Secondly, in this study, although CVD diagnosis depends on self-reporting, this method has been widely accepted in population-based research. Previous verification indicates that it has minimal impact on research results (47, 48). Thirdly, despite the use of multivariate adjustment, confounding factors may still bias the results, but the calculated E value indicates the robustness of the results, and the absence of confounding factors is improbable to alter the conclusions of this investigation. Finally, this study utilizes the CHARLS database, focusing on middle-aged and elderly Chinese participants. Therefore, the findings may not be applicable to other ethnicities, age groups, and geographic regions. In the future, we will expand this study to include participants from different ethnicities, age groups, and geographic regions, which will enhance the applicability of our findings to a wider population.

## Conclusion

This study elucidates a marked link between eGDR and CVD in individuals with CKM syndrome stages 0–3, affirming the efficacy of eGDR as a key biomarker for assessing CVD risk. Notably, this connection manifested as a significant negative nonlinear correlation. This indicated that eGDR has the potential to serve as a predictor for CVD risk assessment and has important clinical value in guiding preventive and management strategies in people with CKM syndrome stages 0–3.

## Data Availability

The original contributions presented in the study are included in the article/Supplementary Material, further inquiries can be directed to the corresponding author.

## Supplementary material

Supplementary Figure S1 Flow chart of the participants with both W1 and W3 eGDR data.

Supplementary Figure S2 Association of cumulative eGDR and the risk of CVD in a population with CKM syndrome stages 0–3 using a multivariable-adjusted RCS model. The model was adjusted for gender, age, residence, marital status, education level, smoking status, drinking status, diabetes, dyslipidemia, diabetes medications, dyslipidemia medications, platelets, CRP, BUN, FBG, Scr, HDL-C, UA, BMI, SBP, and DBP.
