# Supplementary material for: Association between estimated glucose disposal rate and incident cardiovascular disease in a population with Cardiovascular-Kidney-Metabolic syndrome stages 0–3: insights from CHARLS
Source: Front Cardiovasc Med. 2025 Feb 24;12:1537774. doi: 10.3389/fcvm.2025.1537774 (PMC11891229; doi:10.3389/fcvm.2025.1537774)
Supplement: Supplementary Figure S2 — Association of cumulative eGDR and the risk of CVD in a population with CKM syndrome stages 0–3 using a multivariable-adjusted RCS model. The model was adjusted for gender, age, residence, marital status, education level, smoking status, drinking status, diabetes, dyslipidemia, diabetes medications, dyslipidemia medications, platelets, CRP, BUN, FBG, Scr, HDL-C, UA, BMI, SBP, and DBP. [file Datasheet1.zip › Table S1.pdf]

Table S1 Distribution of variables with missing data

variables	Number of Missi	Missing proportio
Cancer	30	0.47%
Lunge disease	22	0.35%
Liver disease	52	0.82%
BMI	69	1.09%
PLT	127	2.00%
BUN	115	1.81%
FBG	131	2.06%
SCR	132	2.08%
TC	121	1.90%
TG	119	1.87%
HDL-C	114	1.79%
LDL-C	130	2.04%
CRP	114	1.79%
UA	114	1.79%
Height	43	0.68%
Weight	36	0.57%
SBP	98	1.54%
DBP	100	1.57%
Smoking status	19	0.30%
Drinking status	4	0.06%

This file has been converted from its original format for security purposes. Please use C704681C14978 as a reference.
