# Supplementary material for: Association between estimated glucose disposal rate and incident cardiovascular disease in a population with Cardiovascular-Kidney-Metabolic syndrome stages 0–3: insights from CHARLS
Source: Front Cardiovasc Med. 2025 Feb 24;12:1537774. doi: 10.3389/fcvm.2025.1537774 (PMC11891229; doi:10.3389/fcvm.2025.1537774)
Supplement: Supplementary Figure S2 — Association of cumulative eGDR and the risk of CVD in a population with CKM syndrome stages 0–3 using a multivariable-adjusted RCS model. The model was adjusted for gender, age, residence, marital status, education level, smoking status, drinking status, diabetes, dyslipidemia, diabetes medications, dyslipidemia medications, platelets, CRP, BUN, FBG, Scr, HDL-C, UA, BMI, SBP, and DBP. [file Datasheet1.zip › Table S2.pdf]

Table S2 Variance inflation factor and tolerance

Term	VIF	VIF_CI_low	VIF_CI_high	SE_factor	Tolerance	Tolerance_CI_low	Tolerance_CI_high
Gender	4.589753	4.395510	4.795108	2.142371	0.21787665	0.20854588	0.2275049
Age	1.974834	1.905384	2.049612	1.405288	0.50637159	0.48789733	0.52482846
Residence	1.111009	1.084837	1.145256	1.054044	0.90008259	0.87316718	0.92179779
Marital status	1.171916	1.142035	1.208083	1.082551	0.85330370	0.82775789	0.87562999
Education level	1.318494	1.280917	1.361097	1.148257	0.75844127	0.73470169	0.78069067
Smoking status	1.085042	1.060768	1.119011	1.041653	0.92162349	0.89364635	0.94271282
Drinking status	1.113934	1.087565	1.148243	1.055431	0.89771933	0.87089550	0.91948526
Diabetes	1.717382	1.660310	1.779388	1.310489	0.58228156	0.56199103	0.60229731
Dyslipidemia	1.693822	1.637886	1.754663	1.301469	0.59038085	0.56990996	0.61054323
Lung disease	1.050967	1.030057	1.086425	1.025167	0.95150435	0.92044987	0.97082022
Liver disease	1.025624	1.009407	1.069799	1.012731	0.97501610	0.93475507	0.99068074
Cancer	1.009704	1.000746	1.126271	1.004840	0.99038898	0.88788608	0.99925475
Hypertension medications	1.440702	1.397062	1.489138	1.200292	0.69410620	0.67152946	0.71578793
Diabetes medications	1.425518	1.382624	1.473220	1.193951	0.70149934	0.67878502	0.72326229
Dyslipidemia medications	1.213876	1.181697	1.251754	1.101760	0.82380750	0.79887901	0.84624086
Platelet	1.060053	1.038078	1.094710	1.029589	0.94334916	0.91348432	0.96331876
BUN	1.163879	1.134455	1.199742	1.078832	0.85919587	0.83351244	0.88148060
FBG	1.703034	1.646653	1.764330	1.305003	0.58718734	0.56678727	0.60729237
Scr	4.379446	4.195145	4.574378	2.092713	0.22833939	0.21860898	0.23837078
TC	15.250156	14.547503	15.989253	3.905145	0.06557310	0.06254201	0.06874032
TG	8.136561	7.773086	8.519541	2.852466	0.12290205	0.11737721	0.12864903
HDL-C	3.366108	3.230157	3.510348	1.834696	0.29707896	0.28487206	0.30958250
LDL-C	12.809288	12.223013	13.426188	3.579007	0.07806835	0.07448130	0.08181289
CRP	1.045205	1.025079	1.081483	1.022353	0.95674995	0.92465624	0.97553462
UA	1.385184	1.344280	1.430947	1.176938	0.72192586	0.69883783	0.74389254

This file has been converted from its original format for security purposes. Please use C6F40A23F6383 as a reference.

Term	VIF	VIF_CI_low	VIF_CI_high	SE_factor	Tolerance	Tolerance_CI_low	Tolerance_CI_high
eGFR	4.287334	4.107427	4.477657	2.070588	0.23324518	0.22333110	0.24346141
Height	1.931862	1.864474	2.004503	1.389914	0.51763526	0.49887675	0.53634420
Weight	1.964124	1.895188	2.038368	1.401472	0.50913292	0.49058849	0.52765224
BMI.	1.559000	1.509588	1.613203	1.248599	0.64143679	0.61988466	0.66243239
MetS	1.813965	1.752242	1.880753	1.346835	0.55127856	0.53170192	0.57069758
SBP	2.614318	2.514265	2.720981	1.616885	0.38250896	0.36751445	0.39773053
DBP	2.217115	2.136057	2.303957	1.488998	0.45103653	0.43403583	0.46815231
eGDR	2.213079	2.132214	2.299720	1.487642	0.45185915	0.43483560	0.46899611

This file has been converted from its original format for security purposes. Please use C6F40A23F6383 as a reference.
