# Supplementary material for: Association between estimated glucose disposal rate and incident cardiovascular disease in a population with Cardiovascular-Kidney-Metabolic syndrome stages 0–3: insights from CHARLS
Source: Front Cardiovasc Med. 2025 Feb 24;12:1537774. doi: 10.3389/fcvm.2025.1537774 (PMC11891229; doi:10.3389/fcvm.2025.1537774)
Supplement: Supplementary Figure S2 — Association of cumulative eGDR and the risk of CVD in a population with CKM syndrome stages 0–3 using a multivariable-adjusted RCS model. The model was adjusted for gender, age, residence, marital status, education level, smoking status, drinking status, diabetes, dyslipidemia, diabetes medications, dyslipidemia medications, platelets, CRP, BUN, FBG, Scr, HDL-C, UA, BMI, SBP, and DBP. [file Datasheet1.zip › Table S3.pdf]

Table S3 Association between the cumulative eGDR and CVD incidence in a population with CKM syndrome stages 0-3

**Characteristic**	**Event, n**	**Model 1**		**Model 2**		**Model 3**	
		**HR (95% CI)**	**p-value**	**HR (95% CI)**	**p-value**	**HR (95% CI)**	**p-value**
Cumulative eGDR (per 1 unit)	794	0.94(0.93, 0.95)	< 0.001	0.95(0.94, 0.96)	< 0.001	0.95(0.94, 0.97)	< 0.001
Cumulative eGDR quartile							
Q1	291	Ref		Ref		Ref	
Q2	207	0.67(0.56, 0.81)	< 0.001	0.67(0.56, 0.81)	< 0.001	0.71(0.59, 0.87)	< 0.001
Q3	169	0.54(0.45, 0.65)	< 0.001	0.56(0.46, 0.68)	< 0.001	0.62(0.50, 0.78)	< 0.001
Q4	127	0.39(0.32, 0.49)	< 0.001	0.41(0.33, 0.51)	< 0.001	0.44(0.34, 0.57)	< 0.001
P for trend			< 0.001		< 0.001		< 0.001

HR = Hazard Ratio, CI = Confidence Interval

Model 1: unadjusted for any covariates.

Model 2: adjusted for gender, age, residence, marital status, education level, smoking status, and drinking status.

Model 3: adjusted for gender, age, residence, marital status, education level, smoking status, drinking status, diabetes, dyslipidemia, diabetes medications, dyslipidemia medications, platelets, CRP, BUN, FBG, Scr, HDL-C, UA, BMI, SBP, and DBP.

This file has been converted from its original format for security purposes. Please use C261476A3EE97 as a reference.
