# Supplementary material for: Association between estimated glucose disposal rate and incident cardiovascular disease in a population with Cardiovascular-Kidney-Metabolic syndrome stages 0–3: insights from CHARLS
Source: Front Cardiovasc Med. 2025 Feb 24;12:1537774. doi: 10.3389/fcvm.2025.1537774 (PMC11891229; doi:10.3389/fcvm.2025.1537774)
Supplement: Supplementary Figure S2 — Association of cumulative eGDR and the risk of CVD in a population with CKM syndrome stages 0–3 using a multivariable-adjusted RCS model. The model was adjusted for gender, age, residence, marital status, education level, smoking status, drinking status, diabetes, dyslipidemia, diabetes medications, dyslipidemia medications, platelets, CRP, BUN, FBG, Scr, HDL-C, UA, BMI, SBP, and DBP. [file Datasheet1.zip › Table S4.pdf]

Table S4 Baseline characteristics stratified by eGDR (defined hypertension based on 130/80 mmHg) quartiles

Characteristic	eGDR Quartiles					p-value
	**Overall**	**Q1**	**Q2**	**Q3**	**Q4**	
No. of subjects	6,359	1,586	1,593	1,591	1,589	
Gender						<0.001
Female	3,461 (54.43%)	878 (55.36%)	846 (53.11%)	789 (49.59%)	948 (59.66%)	
Male	2,898 (45.57%)	708 (44.64%)	747 (46.89%)	802 (50.41%)	641 (40.34%)	
Age, year	59.61 ± 9.49	60.14 ± 9.16	60.40 ± 9.64	61.38 ± 10.02	56.51 ± 8.32	<0.001
Residence						<0.001
Rural	4,140 (65.10%)	930 (58.64%)	1,016 (63.78%)	1,143 (71.84%)	1,051 (66.14%)	
Urban	2,219 (34.90%)	656 (41.36%)	577 (36.22%)	448 (28.16%)	538 (33.86%)	
Marital status						<0.001
Marred	831 (13.07%)	191 (12.04%)	232 (14.56%)	276 (17.35%)	132 (8.31%)	
Other	5,528 (86.93%)	1,395 (87.96%)	1,361 (85.44%)	1,315 (82.65%)	1,457 (91.69%)	
Education level						<0.001
No formal education	3,164 (49.76%)	762 (48.05%)	775 (48.65%)	869 (54.62%)	758 (47.70%)	
Primary school	1,378 (21.67%)	343 (21.63%)	359 (22.54%)	358 (22.50%)	318 (20.01%)	
Middle school	1,224 (19.25%)	321 (20.24%)	304 (19.08%)	256 (16.09%)	343 (21.59%)	
High school or above	593 (9.33%)	160 (10.09%)	155 (9.73%)	108 (6.79%)	170 (10.70%)	
Smoking status						<0.001
Never	3,907 (61.44%)	1,029 (64.88%)	977 (61.33%)	883 (55.50%)	1,018 (64.07%)	
Former	529 (8.32%)	168	135 (8.47%)	125 (7.86%)	101 (6.36%)	

This file has been converted from its original format for security purposes. Please use CC52309A41AC2 as a reference.

Characteristic	eGDR Quartiles					p-value
	Overall	Q1	Q2	Q3	Q4	
		(10.59%)				
Current	1,923 (30.24%)	389 (24.53%)	481 (30.19%)	583 (36.64%)	470 (29.58%)	
Drinking status						<0.001
Never	3,894 (61.24%)	959 (60.47%)	955 (59.95%)	919 (57.76%)	1,061 (66.77%)	
Former	509 (8.00%)	166 (10.47%)	135 (8.47%)	127 (7.98%)	81 (5.10%)	
Current	1,956 (30.76%)	461 (29.07%)	503 (31.58%)	545 (34.26%)	447 (28.13%)	
Hypertension	4,608 (72.46%)	1,584 (99.87%)	1,588 (99.69%)	1,355 (85.17%)	81 (5.10%)	<0.001
Diabetes	1,393 (21.91%)	525 (33.10%)	244 (15.32%)	262 (16.47%)	362 (22.78%)	<0.001
Dyslipidemia	3,653 (57.45%)	1,070 (67.47%)	824 (51.73%)	665 (41.80%)	1,094 (68.85%)	<0.001
Lung disease	559 (8.79%)	131 (8.26%)	137 (8.60%)	169 (10.62%)	122 (7.68%)	0.021
Liver disease	174 (2.74%)	45 (2.84%)	44 (2.76%)	33 (2.07%)	52 (3.27%)	0.221
Cancer	52 (0.82%)	17 (1.07%)	11 (0.69%)	9 (0.57%)	15 (0.94%)	0.371
Hypertension medications	1,351 (21.25%)	646 (40.73%)	434 (27.24%)	253 (15.90%)	18 (1.13%)	<0.001
Diabetes medications	262 (4.12%)	150 (9.46%)	29 (1.82%)	56 (3.52%)	27 (1.70%)	<0.001
Dyslipidemia medications	283 (4.45%)	141 (8.89%)	66 (4.14%)	29 (1.82%)	47 (2.96%)	<0.001
Platelets, (10^9/L)	213.78 ± 72.61	216.53 ± 70.49	213.66 ± 76.50	210.49 ± 70.95	214.44 ± 72.26	0.128
CRP, mg/dl	1.13 (0.59, 2.39)	1.55 (0.83, 3.02)	1.08 (0.61, 2.35)	0.91 (0.51, 2.00)	0.95 (0.52, 2.15)	<0.001
BUN, mg/dl	15.64 ± 4.46	15.73 ± 4.35	15.68 ± 4.48	16.00 ± 4.76	15.18 ± 4.19	<0.001
Scr, mg/dl	0.76 (0.66, 0.88)	0.77 (0.66, 0.90)	0.77 (0.67, 0.89)	0.76 (0.66, 0.88)	0.73 (0.63, 0.85)	<0.001
FBG, mg/dl	104.22 (95.58, 116.73)	109.17 (99.36, 129.92)	102.96 (95.22, 113.94)	102.06 (94.50, 112.32)	103.32 (94.68, 115.74)	<0.001
TC, mg/dl	195.03 ±	202.27 ±	197.94 ±	190.72 ±	189.19 ±	<0.00

This file has been converted from its original format for security purposes. Please use CC52309A41AC2 as a reference.

Characteristic	eGDR Quartiles					p-value
	Overall	Q1	Q2	Q3	Q4	
	39.63	41.71	38.38	36.43	40.40	1
TG, mg/dL	122.13 (83.19, 175.23)	138.06 (97.35, 199.13)	112.39 (80.54, 163.73)	99.12 (69.92, 146.02)	139.83 (94.69, 183.20)	<0.001
HDL-C, mg/dl	48.55 ± 14.72	45.06 ± 13.00	50.67 ± 14.62	54.27 ± 16.64	44.19 ± 11.80	<0.001
LDL-C, mg/dl	116.56 ± 36.54	121.37 ± 39.19	119.41 ± 35.70	112.29 ± 34.78	113.18 ± 35.53	<0.001
HbA1c, %	5.10 (4.90, 5.50)	5.40 (5.10, 5.90)	5.10 (4.90, 5.40)	5.10 (4.80, 5.30)	5.10 (4.80, 5.40)	<0.001
UA, mg/dL	4.51 ± 1.27	4.74 ± 1.34	4.56 ± 1.24	4.42 ± 1.24	4.31 ± 1.23	<0.001
eGFR, mL/min/1.73m2	117.96 (101.98, 135.31)	114.99 (99.45, 131.17)	116.42 (101.42, 133.75)	118.51 (102.23, 135.95)	121.53 (104.79, 139.34)	<0.001
Height, m	1.58 ± 0.09	1.59 ± 0.10	1.57 ± 0.10	1.57 ± 0.09	1.57 ± 0.09	<0.001
Weight, kg	59.54 ± 11.74	68.73 ± 11.19	58.99 ± 9.03	53.41 ± 10.52	57.04 ± 10.28	<0.001
Waist, cm	85.32 ± 12.43	97.15 ± 6.80	86.46 ± 3.81	78.87 ± 9.19	78.81 ± 15.77	<0.001
BMI, kg/m^2	23.53 (21.23, 26.16)	27.04 (25.13, 29.07)	23.60 (22.08, 25.19)	21.09 (19.34, 22.91)	22.85 (20.90, 24.71)	<0.001
MetS	2,257 (35.49%)	879 (55.42%)	482 (30.26%)	302 (18.98%)	594 (37.38%)	<0.001
SBP	135.43 ± 21.08	144.49 ± 18.97	143.23 ± 18.15	138.87 ± 19.58	115.13 ± 11.73	<0.001
DBP	78.33 ± 11.84	83.19 ± 10.82	82.53 ± 10.75	79.35 ± 10.98	68.23 ± 7.95	<0.001
CKM stage						<0.001
Stage 0	107 (1.68%)	0 (0.00%)	0 (0.00%)	0 (0.00%)	107 (6.73%)	
Stage 1	280 (4.40%)	0 (0.00%)	0 (0.00%)	22 (1.38%)	258 (16.24%)	
Stage 2	5,205 (81.85%)	1,307 (82.41%)	1,435 (90.08%)	1,441 (90.57%)	1,022 (64.32%)	
Stage 3	767 (12.06%)	279 (17.59%)	158 (9.92%)	128 (8.05%)	202 (12.71%)	

This file has been converted from its original format for security purposes. Please use CC52309A41AC2 as a reference.
