# Supplementary material for: Association between estimated glucose disposal rate and incident cardiovascular disease in a population with Cardiovascular-Kidney-Metabolic syndrome stages 0–3: insights from CHARLS
Source: Front Cardiovasc Med. 2025 Feb 24;12:1537774. doi: 10.3389/fcvm.2025.1537774 (PMC11891229; doi:10.3389/fcvm.2025.1537774)
Supplement: Supplementary Figure S2 — Association of cumulative eGDR and the risk of CVD in a population with CKM syndrome stages 0–3 using a multivariable-adjusted RCS model. The model was adjusted for gender, age, residence, marital status, education level, smoking status, drinking status, diabetes, dyslipidemia, diabetes medications, dyslipidemia medications, platelets, CRP, BUN, FBG, Scr, HDL-C, UA, BMI, SBP, and DBP. [file Datasheet1.zip › Table S6.pdf]

Table S6 Baseline characteristics stratified by eGDR quartiles after excluding participants with any missing values

Characteristic	eGDR Quartiles					p-value
	Overall	Q1	Q2	Q3	Q4	
No. of subjects	5,850	1,462	1,464	1,459	1,465	
Gender						0.003
Male	2,667 (45.59%)	622 (42.54%)	717 (48.98%)	645 (44.21%)	683 (46.62%)	
Female	3,183 (54.41%)	840 (57.46%)	747 (51.02%)	814 (55.79%)	782 (53.38%)	
Age, year	59.54 ± 9.44	60.93 ± 9.28	61.87 ± 9.72	57.57 ± 8.84	57.80 ± 9.13	<0.001
Residence						<0.001
Rural	3,812 (65.16%)	862 (58.96%)	1,001 (68.37%)	915 (62.71%)	1,034 (70.58%)	
Urban	2,038 (34.84%)	600 (41.04%)	463 (31.63%)	544 (37.29%)	431 (29.42%)	
Marital status						<0.001
Marred	5,121 (87.54%)	1,279 (87.48%)	1,206 (82.38%)	1,329 (91.09%)	1,307 (89.22%)	
Other	729 (12.46%)	183 (12.52%)	258 (17.62%)	130 (8.91%)	158 (10.78%)	
Education level						<0.001
No formal education	2,899 (49.56%)	723 (49.45%)	793 (54.17%)	659 (45.17%)	724 (49.42%)	
Primary school	1,272 (21.74%)	313 (21.41%)	328 (22.40%)	313 (21.45%)	318 (21.71%)	
Middle school	1,135 (19.40%)	285 (19.49%)	251 (17.14%)	319 (21.86%)	280 (19.11%)	
High school or above	544 (9.30%)	141 (9.64%)	92 (6.28%)	168 (11.51%)	143 (9.76%)	
Smoking status						<0.001
Never	3,597 (61.49%)	958 (65.53%)	848 (57.92%)	931 (63.81%)	860 (58.70%)	
Former	486 (8.31%)	153 (10.47%)	120 (8.20%)	130 (8.91%)	83 (5.67%)	
Current	1,767 (30.21%)	351 (24.01%)	496 (33.88%)	398 (27.28%)	522 (35.63%)	
Drinking status						<0.001
Never	3,570 (61.03%)	881 (60.26%)	850 (58.06%)	925 (63.40%)	914 (62.39%)	

This file has been converted from its original format for security purposes. Please use C2C7F0355036A as a reference.

Characteristic	eGDR Quartiles					p-value
	**Overall**	**Q1**	**Q2**	**Q3**	**Q4**	
Former	473 (8.09%)	164 (11.22%)	136 (9.29%)	93 (6.37%)	80 (5.46%)	
Current	1,807 (30.89%)	417 (28.52%)	478 (32.65%)	441 (30.23%)	471 (32.15%)	
Hypertension	2,937 (50.21%)	1,455 (99.52%)	1,341 (91.60%)	97 (6.65%)	44 (3.00%)	<0.001
Diabetes	1,164 (19.90%)	407 (27.84%)	257 (17.55%)	287 (19.67%)	213 (14.54%)	<0.001
Dyslipidemia	3,293 (56.29%)	960 (65.66%)	666 (45.49%)	902 (61.82%)	765 (52.22%)	<0.001
Lung disease	509 (8.70%)	117 (8.00%)	149 (10.18%)	108 (7.40%)	135 (9.22%)	0.037
Liver disease	162 (2.77%)	37 (2.53%)	39 (2.66%)	50 (3.43%)	36 (2.46%)	0.356
Cancer	47 (0.80%)	15 (1.03%)	8 (0.55%)	12 (0.82%)	12 (0.82%)	0.545
Hypertension medications	1,238 (21.16%)	742 (50.75%)	453 (30.94%)	27 (1.85%)	16 (1.09%)	<0.001
Diabetes medications	238 (4.07%)	119 (8.14%)	57 (3.89%)	47 (3.22%)	15 (1.02%)	<0.001
Dyslipidemia medications	258 (4.41%)	135 (9.23%)	54 (3.69%)	42 (2.88%)	27 (1.84%)	<0.001
Platelets, (10^9/L)	212.99 ± 73.02	216.03 ± 72.23	210.07 ± 75.83	212.06 ± 71.44	213.81 ± 72.44	0.151
CRP, mg/dl	1.09 (0.58, 2.29)	1.55 (0.81, 2.96)	1.02 (0.55, 2.22)	1.08 (0.60, 2.14)	0.81 (0.46, 1.75)	0.003
BUN, mg/dl	15.63 ± 4.48	15.64 ± 4.41	15.95 ± 4.77	15.49 ± 4.44	15.43 ± 4.28	0.008
Scr, mg/dl	0.76 (0.64, 0.88)	0.77 (0.67, 0.90)	0.76 (0.67, 0.89)	0.75 (0.63, 0.88)	0.75 (0.63, 0.86)	<0.001
FBG, mg/dl	103.86 (95.58, 116.46)	107.64 (98.50, 122.76)	102.78 (94.86, 114.53)	104.22 (95.76, 117.00)	101.52 (93.96, 111.60)	<0.001
TC, mg/dl	194.78 ± 39.03	201.38 ± 38.93	194.56 ± 36.48	194.23 ± 38.69	188.98 ± 40.94	<0.001
TG, mg/dL	120.36 (82.31, 170.80)	134.52 (94.92, 195.59)	103.54 (73.46, 149.57)	129.21 (88.50, 183.20)	112.39 (77.88, 157.53)	<0.001
HDL-C, mg/dl	48.72 ± 14.70	46.11 ± 13.34	53.04 ± 15.64	45.80 ± 13.42	49.92 ± 15.05	<0.001
LDL-C, mg/dl	117.02 ± 35.96	121.73 ± 37.51	116.08 ± 34.05	116.95 ± 35.94	113.34 ± 35.78	<0.001

This file has been converted from its original format for security purposes. Please use C2C7F0355036A as a reference.

Characteristic	eGDR Quartiles					p-value
	Overall	Q1	Q2	Q3	Q4	
HbA1c, %	5.20 (4.90, 5.50)	5.30 (5.00, 5.70)	5.10 (4.80, 5.40)	5.20 (4.90, 5.50)	5.00 (4.80, 5.30)	<0.001
UA, mg/dL	4.49 ± 1.27	4.72 ± 1.32	4.49 ± 1.27	4.48 ± 1.25	4.26 ± 1.21	<0.001
eGFR, mL/min/1.73m^2	117.68 (101.68, 135.81)	113.59 (98.92, 130.13)	115.90 (100.43, 133.95)	119.57 (103.94, 136.56)	121.96 (104.72, 141.12)	<0.001
Height, m	1.58 ± 0.09	1.58 ± 0.09	1.57 ± 0.09	1.59 ± 0.10	1.57 ± 0.09	<0.001
Weight, kg	59.58 ± 11.73	66.90 ± 11.30	55.63 ± 10.07	62.61 ± 10.44	53.22 ± 9.66	<0.001
Waist, cm	85.32 ± 12.52	95.71 ± 6.85	82.23 ± 7.58	89.42 ± 8.14	73.98 ± 13.90	<0.001
BMI, kg/m^2	23.56 (21.24, 26.15)	26.53 (24.51, 28.71)	22.17 (20.44, 24.05)	24.74 (22.94, 26.74)	21.31 (19.74, 22.99)	<0.001
MetS	1,993 (34.07%)	766 (52.39%)	301 (20.56%)	671 (45.99%)	255 (17.41%)	<0.001
SBP, mmHg	135.26 ± 21.15	148.43 ± 19.59	146.85 ± 20.05	123.99 ± 14.04	121.76 ± 13.76	<0.001
DBP, mmhg	78.26 ± 11.92	84.17 ± 11.89	82.83 ± 12.00	73.73 ± 9.03	72.32 ± 9.45	<0.001
CKM stage						<0.001
Stage 0	106 (1.81%)	0 (0.00%)	0 (0.00%)	5 (0.34%)	101 (6.89%)	
Stage 1	273 (4.67%)	0 (0.00%)	8 (0.55%)	127 (8.70%)	138 (9.42%)	
Stage 2	4,977 (85.08%)	1,272 (87.00%)	1,380 (94.26%)	1,181 (80.95%)	1,144 (78.09%)	
Stage 3	494 (8.44%)	190 (13.00%)	76 (5.19%)	146 (10.01%)	82 (5.60%)	

This file has been converted from its original format for security purposes. Please use C2C7F0355036A as a reference.
