# Supplementary material for: Association between estimated glucose disposal rate and incident cardiovascular disease in a population with Cardiovascular-Kidney-Metabolic syndrome stages 0–3: insights from CHARLS
Source: Front Cardiovasc Med. 2025 Feb 24;12:1537774. doi: 10.3389/fcvm.2025.1537774 (PMC11891229; doi:10.3389/fcvm.2025.1537774)
Supplement: Supplementary Figure S2 — Association of cumulative eGDR and the risk of CVD in a population with CKM syndrome stages 0–3 using a multivariable-adjusted RCS model. The model was adjusted for gender, age, residence, marital status, education level, smoking status, drinking status, diabetes, dyslipidemia, diabetes medications, dyslipidemia medications, platelets, CRP, BUN, FBG, Scr, HDL-C, UA, BMI, SBP, and DBP. [file Datasheet1.zip › Table S7.pdf]

Table S7 Association between the eGDR and CVD incidence in a population with CKM syndrome stages 0-3 after excluding participants with any missing values

Characteristic	Event, n	Model 1		Model 2		Model3	
		**HR (95% CI)**	**p-value**	**HR (95% CI)**	**p-value**	**HR (95% CI)**	**p-value**
eGDR (per 1 unit)	1,545	0.88(0.86, 0.90)	< 0.001	0.89(0.87, 0.91)	< 0.001	0.91(0.88, 0.93)	< 0.001
eGDR quartile							
Q1	537	Ref		Ref		Ref	
Q2	386	0.67(0.59, 0.77)	< 0.001	0.68(0.60, 0.78)	< 0.001	0.71(0.62, 0.81)	< 0.001
Q3	336	0.57(0.50, 0.65)	< 0.001	0.59(0.52, 0.68)	< 0.001	0.65(0.55, 0.76)	< 0.001
Q4	286	0.47(0.41, 0.55)	< 0.001	0.50(0.43, 0.58)	< 0.001	0.55(0.47, 0.66)	< 0.001
P for trend			< 0.001		< 0.001		< 0.001

HR = Hazard Ratio, CI = Confidence Interval

Model 1: unadjusted for any covariates.

Model 2: adjusted for gender, age, residence, marital status, education level, smoking status, and drinking status.

Model 3: adjusted for gender, age, residence, marital status, education level, smoking status, drinking status, diabetes, dyslipidemia, diabetes medications, dyslipidemia medications, platelets, CRP, BUN, FBG, Scr, HDL-C, UA, BMI, SBP, and DBP.

This file has been converted from its original format for security purposes. Please use C2F7F0546FA96 as a reference.
