# Supplementary figures and images for: Association between estimated glucose disposal rate and incident cardiovascular disease in a population with Cardiovascular-Kidney-Metabolic syndrome stages 0–3: insights from CHARLS
Source: Front Cardiovasc Med. 2025 Feb 24;12:1537774. doi: 10.3389/fcvm.2025.1537774 (PMC11891229; doi:10.3389/fcvm.2025.1537774)

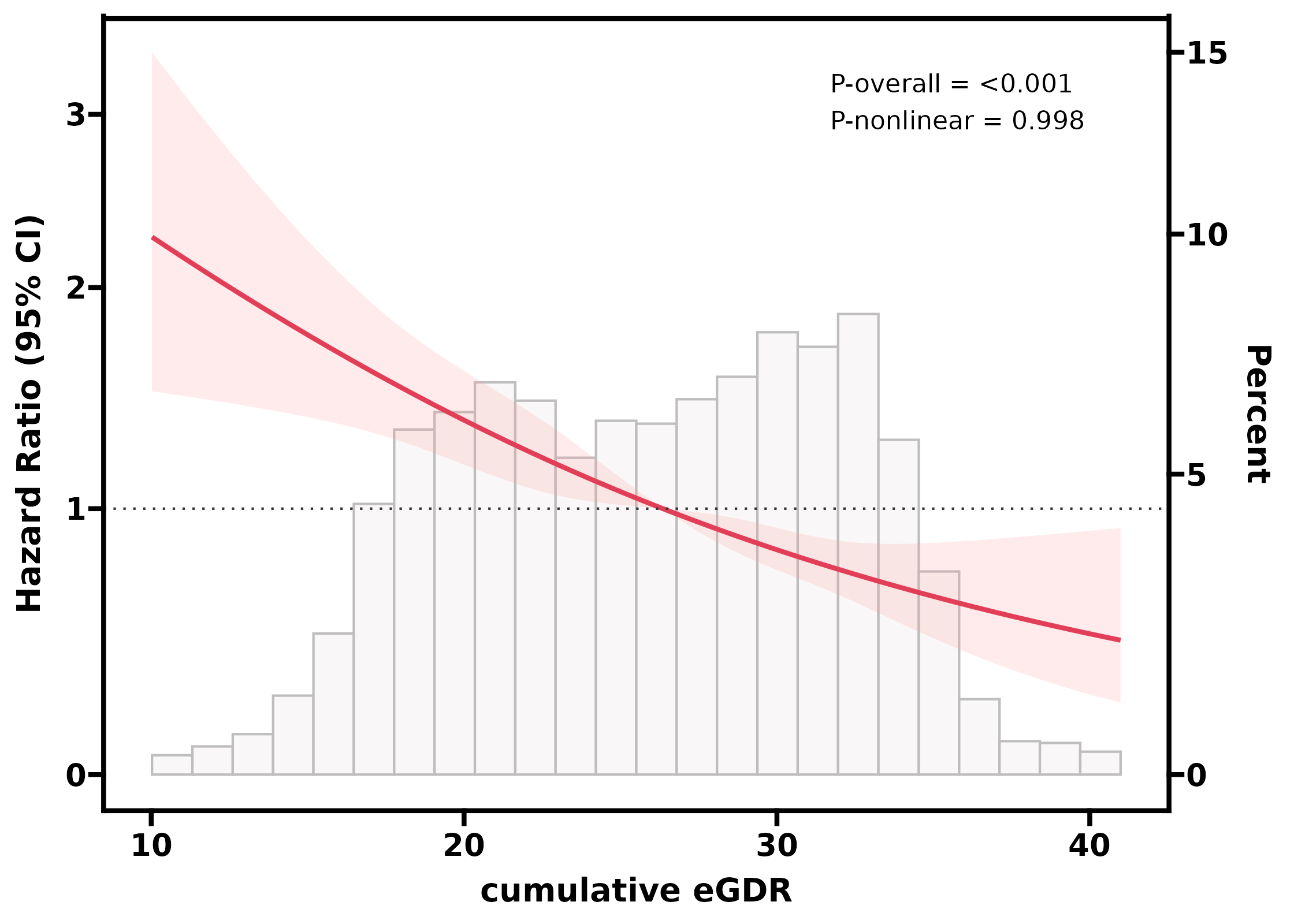

Supplement: Supplementary Figure S2 — Association of cumulative eGDR and the risk of CVD in a population with CKM syndrome stages 0–3 using a multivariable-adjusted RCS model. The model was adjusted for gender, age, residence, marital status, education level, smoking status, drinking status, diabetes, dyslipidemia, diabetes medications, dyslipidemia medications, platelets, CRP, BUN, FBG, Scr, HDL-C, UA, BMI, SBP, and DBP. [file Datasheet1.zip › Figure S2.png]

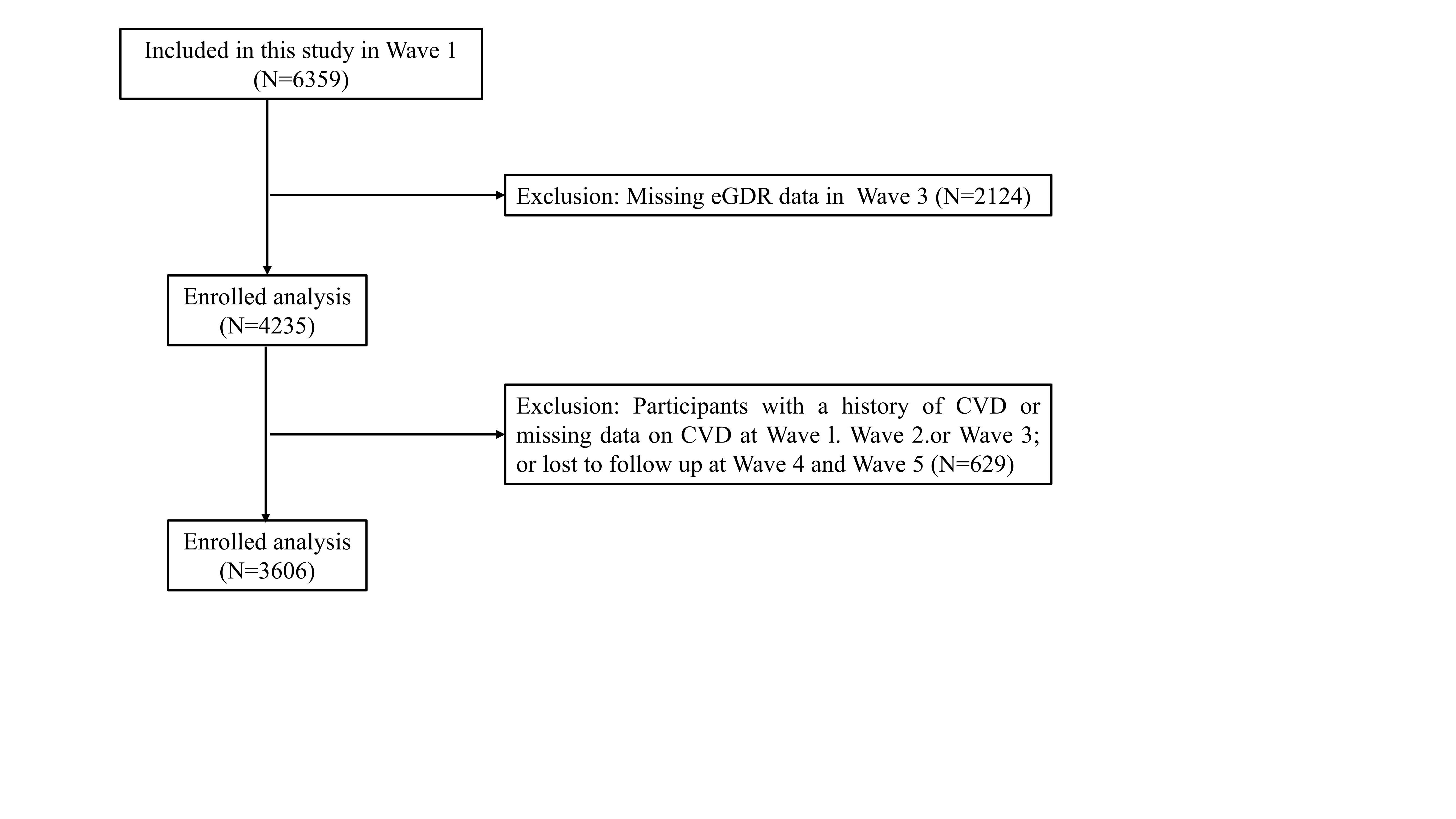

Supplement: Supplementary Figure S2 — Association of cumulative eGDR and the risk of CVD in a population with CKM syndrome stages 0–3 using a multivariable-adjusted RCS model. The model was adjusted for gender, age, residence, marital status, education level, smoking status, drinking status, diabetes, dyslipidemia, diabetes medications, dyslipidemia medications, platelets, CRP, BUN, FBG, Scr, HDL-C, UA, BMI, SBP, and DBP. [file Datasheet1.zip › Figure S1.jpg]
